# A Panoply of Rheumatological Manifestations in Patients with GATA2 Deficiency

**DOI:** 10.1038/s41598-020-64852-1

**Published:** 2020-05-20

**Authors:** Abhimanyu A. Amarnani, Katlin R. Poladian, Beatriz E. Marciano, Janine R. Daub, Sandra G. Williams, Alicia A. Livinski, Amy P. Hsu, Cindy L. Palmer, Cara M. Kenney, Daniele N. Avila, Steven M. Holland, James D. Katz

**Affiliations:** 1 Office of the Clinical Director, Intramural Research Program, National Institute of Arthritis and Musculoskeletal and Skin Diseases, National Institutes of Health, Bethesda, Maryland, USA; 2 SUNY Downstate Health Sciences University, College of Medicine and School of Graduate Studies, Brooklyn, USA; 3 Department of Medicine, Los Angeles County + University of Southern California Medical Center and University of Southern California Keck School of Medicine, Los Angeles, CA 90033, USA; 4 National Institute of Allergy and Infectious Diseases, Intramural Research Program, National Institutes of Health, Bethesda, Maryland, USA; 5 National Institutes of Health Library, Division of Library Services, Office of Research Services, OD, NIH, Bethesda, Maryland, USA; 6 National Cancer Institute, Center for Cancer Research, Office of the Clinical Director, National Institutes of Health, Bethesda, Maryland, USA

**Keywords:** Primary immunodeficiency disorders, Autoimmunity, Rheumatology

## Abstract

Purpose: To characterize rheumatological manifestations of GATA2 deficiency. Methods: Single-center, retrospective review of 157 patients with GATA2 deficiency. Disease course, laboratory results, and imaging findings were extracted. In-person rheumatological assessments were performed on selected, available patients. A literature search of four databases was conducted to identify additional cases. Results: Rheumatological findings were identified in 28 patients, out of 157 cases reviewed (17.8%). Twenty-two of those patients (78.6%) reported symptom onset prior to or in conjunction with the molecular diagnosis of GATA2 deficiency. Notable rheumatological manifestations included: piezogenic pedal papules (PPP), joint hyperextensibility, early onset osteoarthritis, ankylosing spondylitis, and seronegative erosive rheumatoid arthritis. In peripheral blood of patients with rheumatological manifestations and GATA2 deficiency, CD4+ CD3+ helper T cells and naïve CD3+ CD4+ CD62L+ CD45RA+ helper T cell subpopulation fractions were significantly lower, while CD8+ cytotoxic T cell fractions were significantly higher, compared to those without rheumatological manifestations and with GATA2 deficiency. No changes in CD19, CD3, or NK populations were observed. Conclusion: GATA2 deficiency is associated with a broad spectrum of rheumatological disease manifestations. Low total helper T lymphocyte proportions and low naïve helper T cell proportions are associated with those most at risk of overt rheumatological manifestations. Further, PPP and joint hyperextensibility may explain some of the nonimmunologically-mediated joint problems encountered in patients with GATA2 deficiency. This catalogue suggests that rheumatological manifestations and immune dysregulation are relatively common in GATA2 deficiency.

## Introduction

GATA2 is a zinc finger transcription factor that plays a critical role in hematopoietic lineage commitment. Haploinsufficiency of GATA2 underlies five distinct syndromes: (1) Mono-cytopenia and non-tuberculous mycobacterial infection (MonoMAC); (2) Dendritic cell, monocyte, B, and natural killer lymphopenia (DCML); (3) Familial myelodysplasia (MDS)/acute myelogenous leukemia (AML); (4) Emberger syndrome (primary lymphedema with MDS); and (5) classical NK cell deficiency^1–3^. The definitive treatment strategy for patients with GATA2 deficiency is nonmyeloablative hematopoietic stem cell transplantation, which has been shown to successfully reconstitute cell population deficiencies and reverse myelodysplastic and infectious phenotypes^4^.

While the myelodysplastic and infectious disease manifestations in patients with GATA2 deficiency have been well described, only a handful of case reports and case series^5–11^ have reported rheumatological manifestations in patients with GATA2 deficiency. Specifically, erythema nodosum^6,7,10,12^, panniculitis^6,7,9,12–14^, primary biliary cirrhosis^6^, hemophagocytic lymphohistiocytosis-like disease^11^, and uncharacterized arthralgias^5,7,14^ were previously described. Since immune system dysregulation secondary to GATA2 deficiency underlies infectious and myelodysplastic syndromes, and immune system dysregulation is also central to rheumatological disease, we sought to evaluate the extent of rheumatological manifestations in our cohort of patients with GATA2 deficiency.

Through an ongoing National Institutes of Health (NIH) study of GATA2 deficiency, we identified patients with rheumatological findings, specifically patients with definable autoimmune diseases and others with musculoskeletal (MSK) manifestations. Our catalogue of observations in patients with GATA2 deficiency includes observations of manifestations described in the literature, and findings not previously described.

## Results

Through retrospective review of 157 patients identified at the NIH, we identified 28 patients (17.8% of our GATA2 deficiency cohort) with rheumatological findings. Rheumatological manifestations and manifestations of GATA2 deficiency are summarized in Supplemental Table 1. The most notable rheumatological features characterized included: piezogenic pedal papules (PPP), early onset osteoarthritis, ankylosing spondylitis, and seronegative erosive rheumatoid arthritis. Of patients with rheumatological disease manifestations, 22 (79%) reported symptom onset prior to, or in conjunction with, the molecular diagnosis of GATA2 deficiency. Some patients had multiple rheumatological manifestations as indicated in Supplemental Table 1. Rheumatological manifestations that were like those described in previous publications included erythema nodosum (n = 3), panniculitis without underlying non-tuberculous mycobacterial infection (n = 7), hemophagocytic lymphohistiocytosis (n = 1), primary biliary cirrhosis (n = 1), and arthralgias (n = 16). Previously unreported findings were: PPP (n = 6), ankylosing spondylitis (n = 1), seronegative erosive rheumatoid arthritis (n = 1), psoriasis (n = 4), psoriatic arthritis (n = 1), positive lupus anticoagulant with a miscarriage history (n = 1), recurrent pericarditis/serositis (n = 1), Behçet’s disease (n = 1), sicca symptoms (n = 1), livedo reticularis (n = 2), alopecia areata (n = 1), and a reported history of juvenile idiopathic arthritis (JIA) (n = 1) (Supplemental Table 1a).

### Clinical features

#### Arthralgias and synovitis

**Figure 1:**
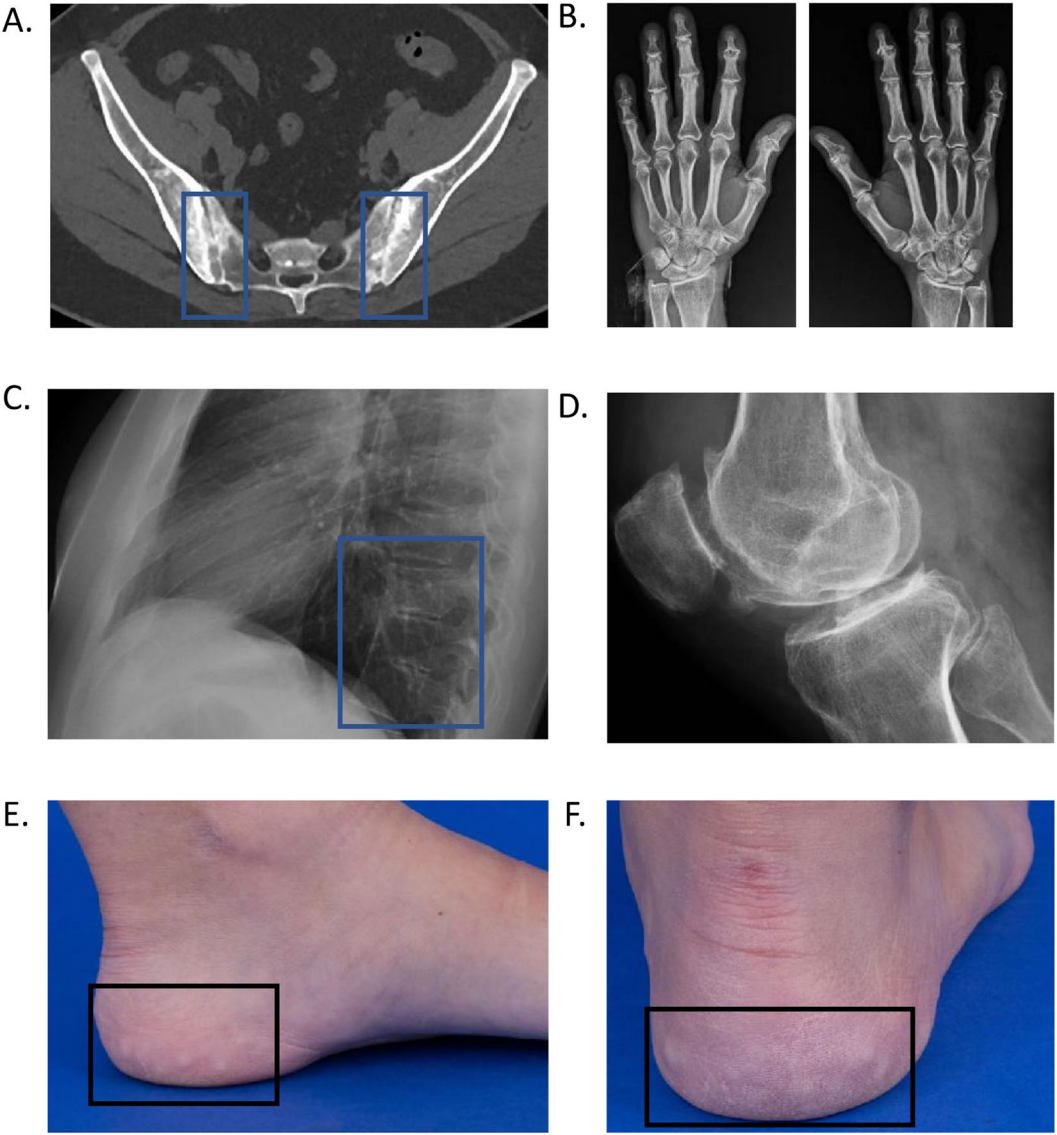
Select abnormalities observed in our Natural History of Disease GATA2 deficiency cohort. (**A**) CT imaging showing sacroiliac irregularity and periarticular sclerosis compatible with sacroiliitis in a 30-year-old male. (**B**) Small juxta-articular erosions involving the distal interphalangeal joints bilaterally, mild-moderate degenerative changes of the radiocarpal joints bilaterally, and tiny juxta-articular erosions involving the proximal interphalangeal joints on the left in a 53-year-old female. (**C**) X-ray imaging showing atraumatic fusion of three thoracic vertebrae in a 20-year-old female. (**D**) Patellofemoral joint x-ray showing spurs and washed out periarticular osteopenia in a 46-year-old male; knee aspiration yielded 60,000 WBCs and no organism or acute gout crystals. (**E,F**) Piezogenic pedal papules observed with weight-bearing, but not when elevated in a 22-year-old female.

Sixteen of our 28 affected patients (57%) had some form of joint complaints. Seven had arthralgias without definitive classification of joint complaints (15.I.1, 16.I.1, 17.I.1, 20.I.1, 21.I.1, 26.I.1, 27.I.1). One 24-year-old male (1.I.1) had a history of morning stiffness, limited forward flexion, night pain, and diminished cervical rotation. Imaging showed sacroiliitis confirming ankylosing spondylitis (Fig. 1A). One 53-year-old female (2.I.1) with a 20-year history of psoriasis and erythema nodosum reported severe joint pain in her shoulders, hands, wrists, and ankles, associated with joint effusions and ambulatory difficulty; she had symmetric polyarticular synovitis and required arthrocentesis, steroid injections, and treatment with sulfasalazine. Bilateral hand radiographs identified radiocarpal and distal interphalangeal joint erosive changes without metacarpophalangeal or proximal interphalangeal joint involvement (Fig. 1B). This patient was clinically reclassified with the diagnosis of psoriatic arthritis. A 36-year-old female (3.I.1) had a history of juvenile idiopathic arthritis (JIA) was diagnosed at age 9 and successfully treated with methotrexate until remission at age 17^15^. Her JIA subtype was unclear from records and it is unclear if her initial diagnosis would meet updated JIA classification guidelines^15^. A 46-year-old male (4.I.1) with a history of uveitis and granulomatous dermatitis reported joint pain beginning at age 29, affecting his left ankle first, and later his wrists, knees, elbows, and shoulders. His clinical diagnosis was seronegative erosive rheumatoid arthritis. His treatment history included methotrexate, etanercept, tocilizumab, abatacept, and certolizumab. He stopped all immunosuppressive treatments due to recurrent pneumonia and cellulitis. Arthrocentesis yielded 16,000 WBCs, 83% neutrophils, 12% lymphocytes, with no organisms identified (including no mycobacteria or fungi) and no crystals. Radiographs of his knees showed spur formation and periarticular osteopenia (Fig. 1D).

#### Piezogenic pedal papules and joint hypermobility

Six patients had PPP (1.I.1, 5.I.1, 6.I.1, 6.I.2, 7.I.1, 7.II.1). One 22-year-old female (5.I.1) with a history of congenital absence of one kidney and cytomegalovirus-induced hemophagocytic lymphohistiocytosis was seen for hand pain. She had no synovitis, tenderness, swelling, or inflammatory findings, but did have numerous PPP, 10-degree hyperextension of the knees bilaterally, and a high arched palate (Fig. 1E,F). A 21-year-old female (6.I.1) had atraumatic fusion of three thoracic vertebrae (Fig. 1C), without back, hip, or groin pain and no morning stiffness or overt inflammatory findings. She also had PPP. Her brother, a 22-year-old (6.I.2) with GATA2 deficiency and osteopenia refractory to calcium and vitamin D supplementation also had PPP and increased range of motion in his shoulders bilaterally. Their mother, who does not have GATA2 deficiency did not have PPP. PPP were also observed in the patient described above with ankylosing spondylitis (1.I.1). Another 36-year-old female with a history of psoriasis also had PPP (7.I.1), along with 10 degrees of hyperextension at her elbows bilaterally, and 15 degrees of hyperextension at her knees bilaterally. Her 16-year old daughter (7.II.1), who also has GATA2 deficiency, was found to have PPP, hypermobile joints, pes planus, chondromalacia patellae, and hypertelorism, but no bifid uvula.

#### Osteopenia and early-onset osteoporosis

One 22-year-old female (8.I.1) with biopsy-defined left leg panniculitis with eosinophils and neutrophils without histologically identifiable microorganisms had a history of right knee swelling with effusion that required arthrocentesis. Pre-transplant CT showed severe cortical bone loss and osteopenia in bilateral femora and tibiae while on 20 mg of prednisone daily.

To further evaluate osteopenia, we reviewed available DEXA scans for all patients with GATA2 deficiency. The age range of the subjects was 15–34 years old, and all DEXA scans available were completed in patients post-transplant. We observed that 8 of 54 patients (14.8%) had lumbar spine scores in the osteoporosis range (<2.5, Z-score). In addition, assessment of bone trabecular-meshwork microarchitecture was accomplished through trabecular bone scores (TBS). TBS are a DEXA-derived measurement that identify fracture risk independent of bone mineral density^16^. Of 30 patients with available TBS, 4 patients had levels indicating intermediate fracture risk (<1.31 (13.3%)), 2 of which also had low lumbar DEXA scans. Four of the patients with osteopenia/osteoporosis had other rheumatological manifestations and are described in Supplemental Table 1 (8.I.1, 14.I.1, 17.I.1, 23.I.1).

#### Psoriasis

Four patients with a history of psoriasis were identified (2.I.1, 7.I.1, 9.I.1, 10.I.1). Two are described above, one as psoriatic arthritis (2.I.1) and one with PPP (7.I.1). Another patient’s psoriasis was successfully treated with ustekinumab without return of symptoms post-transplant (9.I.1). A 54-year-old male (10.I.1) with a history of back pain beginning in his 20s, associated with nighttime awakening and morning stiffness had no sacroiliitis on imaging, but did have a history of psoriasis. Psoriasis treatment included topical steroids, complicated by a history of imiquimod for warts.

Unexplained Multisystem Disease with Evidence of Autoimmunity (Lupus anticoagulant, recurrent pericarditis/serositis, Behçet’s disease, sicca symptoms, and hemophagocytic lymphohistiocytosis).

Both well-defined, and incompletely-defined, autoimmune rheumatological manifestations were observed in six patients: (1) a 52-year-old female (11.I.1) with positive lupus anticoagulant on two instances >12 weeks apart had a history of two miscarriages (Sapporo diagnostic criteria for antiphospholipid syndrome unfulfilled because miscarriage trimesters are unknown), (2) a 45-year-old female (12.I.1) with a 15-year history of recurrent pericarditis/serositis including pericardial effusion with lymphocytic and macrophage predominance, hyperferritinemia, and arthralgias had pulmonary alveolar proteinosis (PAP) despite both a negative infectious workup and a negative autoinflammatory genetic assessment, (3) a 25-year-old female (13.I.1) who was diagnosed at age 11 with painful oral/genital ulcers and polyarticular joint pain satisfying a clinical diagnosis of Behçet’s disease, (4) a 27-year-old female (14.I.1) with livedo reticularis and chronic sicca symptoms, diminished salivary flow, positive ANA, positive anti-proteinase 3, but negative salivary gland biopsy and no notable vasculitis findings, (5) 4.I.1 with history of uveitis, and (5) a 21-year-old male (8.I.1) with a history of hemophagocytic lymphohistiocytosis.

#### Summary of infections, myelodysplastic, and rheumatological clinical features

**Figure 2:**
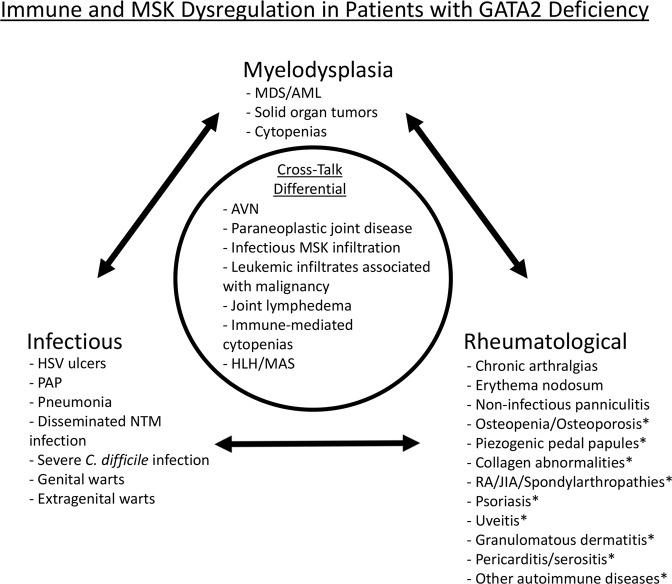
Immune and MSK dysregulation in patients with GATA2 deficiency. Summary of previously published infectious, myelodysplastic, and rheumatological disease manifestations observed in patients with GATA2 deficiency, as well as rheumatological manifestations described herein for the first time (indicated with an asterisk*). MDS - familial myelodysplasia, AML - acute myelogenous leukemia, AVN – avascular necrosis, MSK - musculoskeletal, HLH/MAS - hemophagocytic lymphohistiocytosis/macrophage activation syndrome, HSV – herpes simplex virus, PAP – pulmonary alveolar proteinosis, NTM – nontuberculous mycobacteria, RA – rheumatoid arthritis, JIA – juvenile idiopathic arthritis.

In Fig. 2, we summarize the clinical features related to immune dysregulation that we have observed in our cohort. These include manifestations previously described^1,2,5^. Rheumatological manifestations reported herein for the first time are indicated with an asterisk (*). We also provide a list of differential diagnoses germane to any patient with MSK signs or symptoms in the background of GATA2 deficiency.

### Immunological features

#### Lymphocyte phenotyping

**Figure 3:**
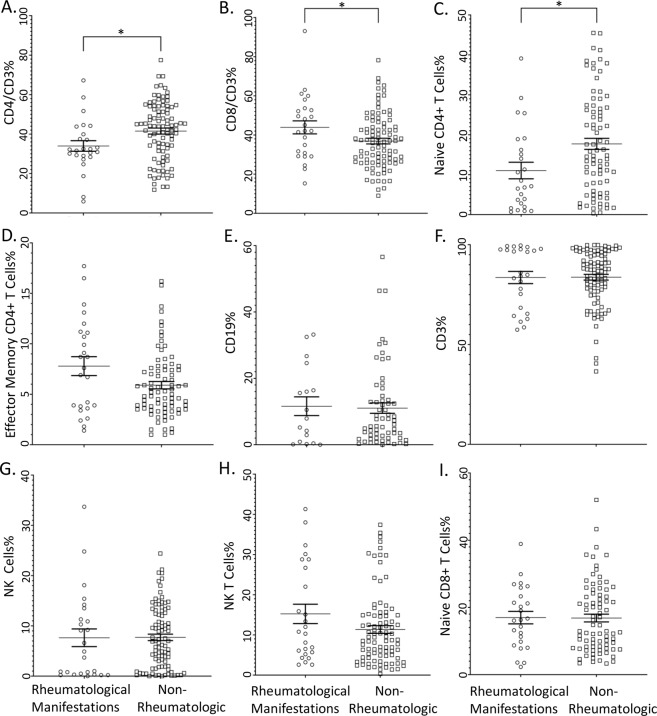
Lymphocyte phenotyping from peripheral blood of patients with GATA2 deficiency. (**A**) CD4+ CD3+ helper T cells proportions. (**B**) CD8+/CD3+ cytotoxic T cell proportions. (**C**) CD3+ CD4+ CD62L+ CD45RA+ True Negative naïve helper T cell subset proportions. (**D**) CD3+ CD4+ CD62L− CD45RA− Effector Memory helper T cell subset proportions. (**E**) CD19+ B cells proportions. (**F**) CD3+ T cell proportions. (**G**) CD16+ and/or CD56+ NK cell proportions. (**H**) CD16+ and/or CD56+, CD3+ NK T cell proportions. (**I**) CD3+ CD8+ CD62L+ CD45RA+ Naïve cytotoxic T cell subset proportions. Box-whisker plot indicates the 10–90% distribution, with outliers identified as scatter dots. *p < 0.05 Mann-Whitney U test. Rheumatologic group (n = 25 of 28 patients) and non-rheumatologic group (n = 95 of 129 patients).

Given the observed rheumatological disease findings in our cohort, we sought to define whether differences in lymphocyte subset proportions were observed between patients with GATA2 deficiency who had rheumatological manifestations and those who did not. We found that CD3+ CD4+ helper T subset proportions were significantly lower in patients with rheumatological manifestations and CD3+ CD8+ cytotoxic T cell proportions were significantly higher (Fig. 3A,B). Further, a significant decrease in naïve helper T cells (CD3+ CD4+ CD62L+ CD45RA+) were significantly lower in patients with rheumatological manifestations, with no difference observed in effector memory helper T cells (CD3+ CD4+ CD62L− CD45RA−) (Fig. 3C,D). No significant differences were observed with CD19+, CD3+, NK cell, NK T cell, or naïve cytotoxic T populations (Fig. 3E–I). No significant differences in effector memory cytotoxic T cells (CD3+ CD8+ CD45RA−CD62L−), central memory-like helper T cells (CD3+ CD4+ CD45RA− CD62L+), central memory-like cytotoxic T cells (CD3+ CD8+ CD45RA− CD62L+), or double negative T cells (CD3+ CD4− CD8−) were observed (data not shown).

### Review of literature

**Figure 4:**
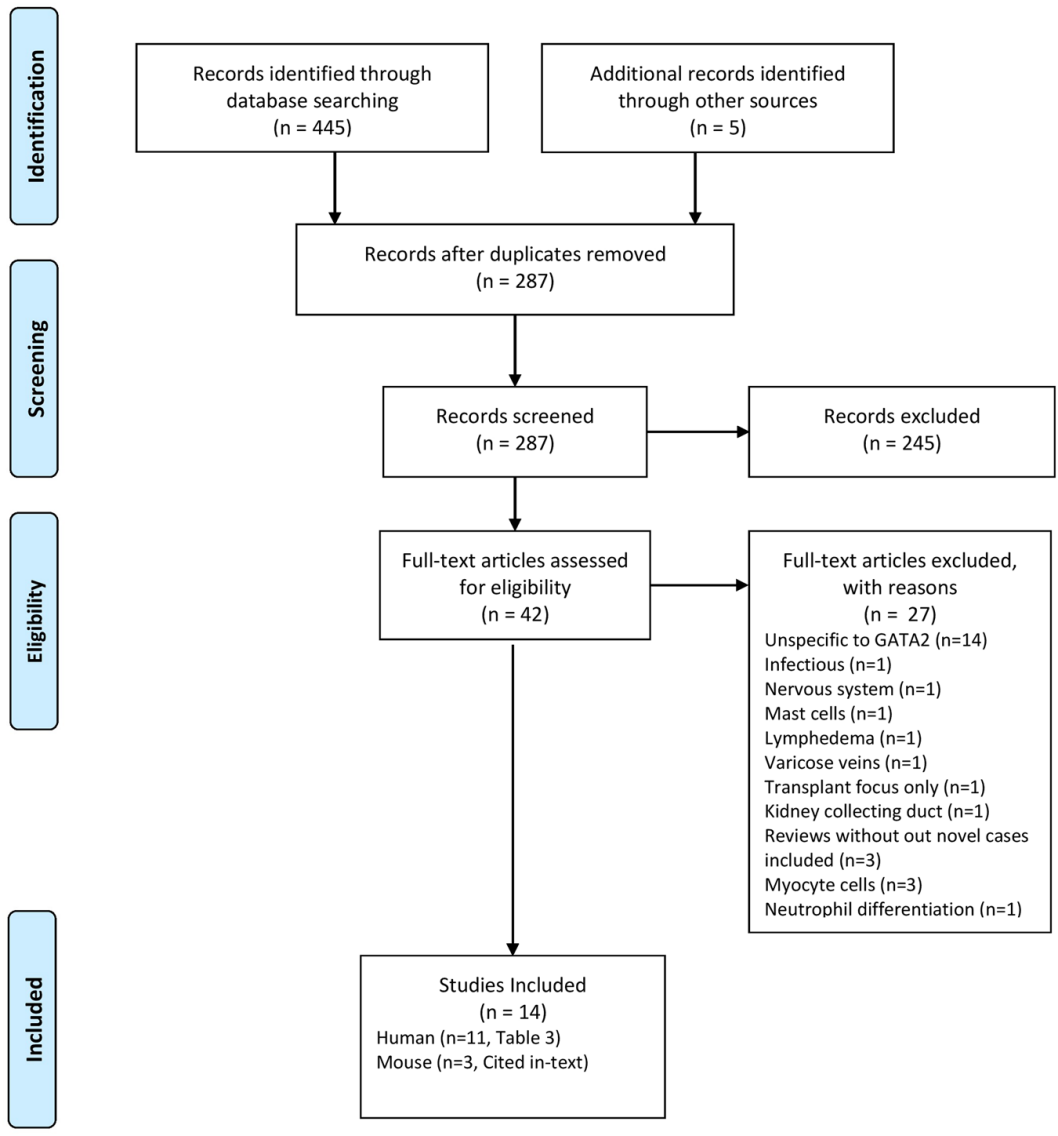
Flow-diagram of literature review. Records were included if they described GATA2 pathology in patients or direct mechanisms in animal models, and were related to inflammation, bone disease, muscle disease, fractures, spondyloarthropathies, rheumatoid arthritis, and other rheumatological manifestations. Only human studies identified are summarized in Supplemental Table 2.

We sought to summarize the literature describing rheumatological manifestations in patients with GATA2 deficiency. Figure 4 shows a flow diagram of the literature review methodology. There were 282 unique records identified (search criteria outlined in Supplemental Methods). Five additional records were included based upon references uncovered during full-text review. Eighteen studies were deemed relevant to rheumatological manifestations or GATA2 mechanisms related to findings observed, inclusive of 11 human (Supplemental Table 2) and three mouse studies.

## Discussion

In this large retrospective study, we have characterized rheumatological manifestations in patients with GATA2 deficiency, specifically identifying musculoskeletal and autoimmune diseases. We have identified cases consistent with previous observations and have also uncovered manifestations that have not previously been reported including PPP, ankylosing spondylitis, psoriasis, psoriatic arthritis, juvenile idiopathic arthritis, seronegative erosive rheumatoid arthritis, positive lupus anticoagulant with a miscarriage history, recurrent pericarditis/serositis, Behçet’s disease, sicca symptoms, livedo reticularis, and alopecia areata. Further, through lymphocyte phenotyping, we identified significant decreases in helper T cells and naïve helper T cell subsets particularly in patients with rheumatological manifestations. These observations indicate important opportunities for primary care providers and rheumatologists to consider GATA2 deficiency in patients presenting with musculoskeletal complaints, infections, myelodysplasia, autoimmune diseases, or lymphedema (Fig. 2). In addition, these observations indicate that metabolic bone turnover, congenital collagen abnormalities, and further analysis of lymphocyte derangements may be fruitful for future investigations in patients with GATA2 deficiency.

This study is limited by its design because it was conducted to solely categorize the full range of our observations to enhance future research into rheumatological consequences associated with GATA2 deficiency. As such, our descriptive, retrospective review methodology precludes statistical inferences based upon our cohort to be compared or generalized to the population in general. For example, while four patients with psoriasis were identified through our retrospective review, the statistical incidence of psoriasis in our cohort compared to the incidence of psoriasis in the general population cannot be accurately determined. Instead, a future dedicated prospective protocol will be able to determine the prevalence of psoriasis in this genetic population.

Our study is also limited in that not all patients identified through retrospective chart review were able to be comprehensively evaluated by a rheumatologist. We identified 28 patients via chart review with rheumatological manifestations and 12 were seen in-person during the study period. As such, we have likely under-ascertained the true magnitude of connective tissue disease burden in this population.

While autosomal dominant inheritance patterns of monocytopenia, early MDS/AML, sensorineural hearing loss, and Emberger syndrome in patients with GATA2 haploinsufficiency have been defined, our study was not designed to comprehensively identify inheritance patterns of rheumatological manifestations. However, while limited, our MSK observations amongst siblings (6.I.1, 6.I.2) and parent-child groups (7.I.1, 7.II.1) support inheritance of at-least some rheumatological manifestations in GATA2 deficiency. In addition, our study was not designed to clarify temporal relationships in patients with GATA2 deficiency. Specifically, while 22 of 28 patients (79%) reported rheumatological symptom onset prior to, or in conjunction with, the molecular diagnosis of GATA2 deficiency, we did not define changes in lymphocyte phenotype at time of rheumatological vs. myelodysplastic vs. infectious symptom onset. Further, our study design does not clarify if definitive treatment for GATA2 deficiency through stem cell transplant cures rheumatological manifestations.

The observation of PPP in several patients with GATA2 deficiency suggests that GATA2 deficiency may be associated with abnormalities in collagen architecture. PPP are classically associated with heritable connective tissue disorders and occur upon weight bearing as fat herniates through a weakened collagen meshwork. Of the six patients identified, a sibling pair with GATA2 deficiency was found to have PPP (6.I.1, 6.I.2), but their mother without GATA2 deficiency did not have this finding. Another patient and her daughter (7.I.1, 7.II.1), both with GATA2 deficiency, had PPP. Although further pedigree data is necessary, the observation of PPP in these families supports the hypothesis that PPP maybe a specific finding in patients with GATA2 deficiency. Furthermore, since three of six patients identified with PPP also were observed to have hypermobile joints (5.I.1, 7.I.1, 7.II.1), this suggests that PPP may be associated with hypermobility of joints, which in turn can lead to early osteoarthritis. This proposed functional mechanism may explain some of the nonimmunologically-mediated joint problems encountered in our cohort, a hypothesis to be further explored in future studies. While our literature review did not identify any studies directly linking GATA2 deficiency with collagen abnormalities, animal models have shown that transcription upregulation of metalloproteinase-2 (MMP-2) in endothelial cells involves GATA2^17^ and MMP-2 can mediate degradation and remodeling of collagen in annulus fibrosus cells at the intervertebral disc^18^.

We identified osteopenia and degenerative joint disease in several patients with GATA2 deficiency, predominantly in the post-transplant period. One pre-transplant patient was incidentally diagnosed by CT scan (8.I.1). Whether this reflects dysregulated bone metabolism or post-bone marrow transplantation osteopenia remains unclear. In support of the former, one study of mesenchymal stem cell-specific GATA2 deficiency in mice demonstrated impaired trabecularization and mechanical strength of bone^19^. In addition, we note that GATA2 plays regulatory roles in association with known bone health modulators, including RANKL, PU.1, and EVI-1^20,21^. Since GATA2 regulates osteoblastogenesis, this warrants further study of bone health in patients with GATA2 deficiency.

We identified four patients with histories of psoriasis. Topical imiquimod (used for recalcitrant warts in GATA2 deficiency) drives the development of psoriasis in a mouse model^22^, and an association of imiquimod and psoriasis has been reported in at least one case report^23^. Therefore, we are scrutinizing our experience with imiquimod in GATA2 deficiency.

Underlying cellular mechanisms were not extensively explored in the present manuscript. Instead, we describe modest associations with clinical lymphocyte phenotyping from peripheral blood. Specifically, we observed that patients with GATA2 deficiency and rheumatological manifestations have decreased CD4+ helper T cell proportions and a decreased naïve helper T cell sub-compartment. These results are consonant with findings in patients with idiopathic CD4 lymphocytopenia, who also have decreased naïve helper T cells^24^.

Others have described changes in B cell subtypes and dendritic cell-regulatory T cell interactions that may also support a connection between GATA2 deficiency and autoimmune disease^7^. An expansion of a small subset of CD38^low^CD21^low^ B cells in patients with GATA2 deficiency was described^14^. This subset is also expanded in the peripheral blood of patients with systemic lupus erythematosus (SLE)^25^, seropositive rheumatoid arthritis (RA)^26^, and Sjögren’s syndrome^27^. Further, in patients with RA, CD21^low^ B cells were identified as a major B cell population in synovial fluid^28^. Also, synovial fluid FcRL+ B cells that produce RANKL stimulate osteoclasts to express lower levels of CD21 than FcRL- B cells^29^. Finally, a subgroup of patients with RA and elevated proportions of peripheral CD21^low^ B cells were shown to have defective ataxia telangiectasia-mutated expression, with associated decreased osteoprotegerin and increased RANKL production^30^. These findings suggest that expansion of CD21 ^low^ B cells may mechanistically contribute to decreased bone density in autoimmune pathologies. In the present study, only eight patients with rheumatological manifestations and 34 without had lymphocyte phenotyping of CD38^low^CD21^low^ B cells available for evaluation. As such,results are underpowered to claim any such association in our cohort. Further prospective evaluation of this B cell subset may clarify if there is a correlation with autoimmune manifestations and bone disease in patients with GATA2 deficiency.

Dendritic cells and regulatory T cells warrant further prospective evaluation. Specifically, dendritic cell deficiency was correlated with depletion of regulatory T cells in patients with GATA2 deficiency^7^, and regulatory T cells play key self-tolerance roles that, when dysregulated, can contribute to many autoimmune diseases including SLE and RA^31^.

In summary, our observations contribute to enhancing an appreciation of the range of rheumatological manifestations associated with GATA2 deficiency. Diagnosis of GATA2 deficiency remains a challenge, and early detection may help direct treatment as well as direct patients to definitive therapy. We describe rheumatological associations, some of which have not previously been reported. Those caring for patients with diagnosed GATA2 deficiency should consider rheumatological disease as a possible contributor to clinical complaints. Moreover, it is essential to appreciate that rheumatological manifestations may precede or overshadow the more notorious GATA2 deficiency associated infectious and myelodysplastic syndromes (Fig. 2)^1,2^.

## Patients and Methods

### Patients

A single-center, retrospective review of 157 patient charts included extracting the disease course, laboratory results, and imaging findings from their medical records. Specifically, all patient charts were reviewed for history of generalized arthralgias, seronegative spondyloarthropathies, rheumatoid arthritis, pediatric inflammatory disease history, erythema nodosum, panniculitis, osteopenia/osteoporosis, psoriasis, granulomatous disease, pericarditis/serositis, and other autoimmune diseases was conducted. Furthermore, laboratory review for anti-nuclear antibodies, rheumatoid factor, and dsDNA results for all patients. A subset of study subjects returning for routine follow-up during the study period of September-November 2019 (n = 12) underwent in-person rheumatological assessments with AA and JDK. Chart review and in-person rheumatological assessments included both patients pre- and post-hematopoietic stem cell transplant.

### Compliance with ethical standards

All patients or their guardians gave written informed consent in accordance with the Declaration of Helsinki for Institutional Review Board. The Institutional Review board of the Division of Allergy and Infectious Diseases, National Institutes of Health, USA approved the study. Protocols included 13-C-0132 “Allogeneic Hematopoietic Stem Cell Transplant for Patients with Mutations in GATA2 or the MonoMAC Syndrome” (NCT01861106) and 13-I-0157 “The Natural History of GATA2 Deficiency and Related Disorders” (NCT01905826). No selection criteria for the specific genetic GATA2 mutation or severity of deficiency were implemented in patient selection. Rheumatological manifestations were classified based on diagnoses indicated in chart review, and through in-person clinical assessments when possible.

### Flow cytometry

Flow cytometry was used for lymphocyte phenotyping from peripheral blood. Frequency for the following cell-type subsets were evaluated from the same flow cytometry studies: B cells (CD19+), T cells (CD3+), T-helper cells (CD4+ CD3+), T-cytotoxic cells (CD8+ CD3+), NK cells (CD16+ and/or CD56+, CD3−), NK T cells (CD16+ and/or CD56+, CD3+), Effector Memory T-helper cells (CD3+ CD4+ CD62L− CD45RA−), Naïve T-helper cells (CD3+ CD4+ CD62L+ CD45RA+), Naïve cytotoxic T cells (CD3+ CD8+ CD62L+ CD45RA+), and Effector Memory CD8+ T cells (CD3+ CD8+ CD62L− CD45RA−). All statistical analyses utilized the Mann-Whitney U, non-parametric test. Supplemental Fig. 1 provides fluorophores and gating strategies used.

### Review of literature

The databases PubMed/MEDLINE (NLM), Embase (Elsevier), Cochrane Library: CENTRAL and Database of Systematic Reviews (Wiley), and Scopus (Elsevier) were searched. Only articles published in English were included; no publication date limit was applied. Conference papers/proceedings/abstracts were excluded, but all other article types were included. Controlled vocabulary terms and keywords related to GATA2 deficiency that linked to spondyloarthropathies, inflammatory bowel disease, osteoarthritis, psoriasis, fractures, back pain, inflammation, and other conditions of interest were used. A review of the bibliographies of included studies was conducted. The final search strategies are provided in Supplemental Methods. Citations were managed and screened in EndNote X9. One author (AA) screened the titles and abstracts and full text to identify studies on GATA2 deficiency and conditions of interest.

## Supplementary information

Supplementary Information.
