# Supplementary material for: A Panoply of Rheumatological Manifestations in Patients with GATA2 Deficiency
Source: Sci Rep. 2020 May 20;10:8305. doi: 10.1038/s41598-020-64852-1 (PMC7239896; doi:10.1038/s41598-020-64852-1)
Supplement: Supplementary file 1 — Supplementary Information. [file 41598_2020_64852_MOESM1_ESM.pdf]

SUPPLEMENTAL MATERIALS AND METHODS

A Panoply of Rheumatological Manifestations in Patients with GATA2 Deficiency

Scientific Reports

Abhimanyu A. Amarnani (1, 2, 3), Katlin R. Poladian (1), Beatriz E. Marciano (4), Janine R. Daub (4), Sandra G. Williams (1), Alicia A. Livinski (5), Amy P. Hsu (4), Cindy L. Palmer (4), Cara M. Kenney (6), Daniele N. Avila (6), Steven M. Holland (4), James D. Katz (1)

Affiliations:
[1]Office of the Clinical Director, Intramural Research Program, National Institute of Arthritis and Musculoskeletal and Skin Diseases, National Institutes of Health, Bethesda, Maryland, USA
[2]SUNY Downstate Health Sciences University, College of Medicine and School of Graduate Studies, Brooklyn, NY, USA
[3]Department of Medicine, Los Angeles County + University of Southern California Medical Center and University of Southern California Keck School of Medicine, Los Angeles, CA 90033, USA
[4]National Institute of Allergy and Infectious Diseases, Intramural Research Program, National Institutes of Health, Bethesda, Maryland, USA
[5]National Institutes of Health Library, Division of Library Services, Office of Research Services, OD, NIH, Bethesda, Maryland, USA
[6]National Cancer Institute, Center for Cancer Research, Office of the Clinical Director, National Institutes of Health, Bethesda, Maryland, USA

Corresponding Author:
Abhimanyu Amarnani, MD, PhD
Room 10N-311A, 10 Center Drive,
National Institutes of Health,
Bethesda, MD 20892-1930, USA
aamarnan@usc.edu
Tel: 301-451-6807

Supplementary Materials and Methods includes four items organized as:

Supplemental Table 1a and 1b. Summary table of 28 patients with rheumatological manifestations and GATA2 deficiency.

Supplemental Table 2. Literature review results.

Supplemental Figure 1. Lymphocyte phenotyping and gating strategy.

Supplemental Methods: Search strategies for literature review

Supplemental Table 1a. Summary table of 28 patients with rheumatological manifestations and GATA2 deficiency.

Patient	Age of Symptom Onset	Age at GATA2 Diagnosis	Age at Transplant	Gender	HLA-B type	Rheumatological Manifestations									
						Arthralgias/Arthritis	PPP (Fig 1e,f)/ Hyperextensibility	Psoriasis	Osteopenia/Early-Onset Osteoporosis	Panniculitis	Livedo Reticularis	Erythema Nodosum	Autoimmune/Inflammatory Manifestations	Other/Descriptions	Serology Results (Max)
1.I.1	24	30	NA	M	44 homozygous	+	+							· Ankylosing Spondylitis<br>· Diminished neck flexion<br>· Modified Schober's 15->17.5 cm<br>· Sacroiliitis (Fig 1a)<br>· Treated with sacroiliac joint corticosteroid injection	· ANA: Neg<br>· RF: <10<br>· dsDNA: Neg<br>· IgA anti:gliadin: 30
2.I.1	33	52	NA	F	41, 51	+		+				+		· Psoriatic arthritis<br>· Took sulfasalazine for arthralgias, shoulder, hands, wrist, ankles, since 30s<br>· Hx of arthrocentesis<br>· Radiographic bilateral, symmetric radiocarpal, DIP erosive changes without significant MCP or PIP involvement (Fig 1b)<br>· Psoriasis/PsA supported<br>· Erythema nodosum<br>· Splenic artery aneurysm	· ANA: 2.5<br>· RF: <15<br>· dsDNA: Neg

3.I.1	9	36	40	F	08,44	+			· History of JIA controlled with MTX<br>· Unknown JIA subtype, nor details regarding initial diagnosis	· ANA: NA<br>· RF: NA<br>· dsDNA: NA
4.I.1	27	45	NA	M	18, 53	+		+	· Seronegative erosive rheumatoid arthritis<br>· Patellofemoral joint x-ray imaging showed spurs and washed out periarticular osteopenic erosions (Fig 1d)<br>· Knee aspiration yielded 16,000 WBCs, no crystals, 83% neutrophils, 12% lymphocytes<br>·Had taken MTX, abatacept, adalimumab, and certolizumab<br>· Granulomatous dermatitis (Biopsy proven)<br>· Uveitis	· ANA: NA<br>· RF: NA<br>· dsDNA: NA
5.I.1	22	22	NA	F	44, 45	+	+		· Hypermobile joints knees, ankles, shoulder<br>· Joint pain with swelling<br>· Congenital absence of one kidney<br>· Hyperferritinemia<br>· Leukopenia/pancytopenia/coagulopathy	· ANA: Neg<br>· RF: <15<br>· dsDNA: Neg
6.I.1	Birth	16	18	F	NA		+		· Atraumatic fusion lumbar vertebrae<br>· Bilateral hip xrays and lumbar spine imaging showed fusion of three thoracic vertebrae and increased trochlear neck length (Fig 1c)	· ANA: Neg<br>· RF: Neg<br>· dsDNA: NA
6.I.2	Unknown	22	23	M	07, 08		+		· Increased shoulder range of motion<br>· DEXA-scan showing osteopenia/osteoporosis, refractory to calcium and vitamin D supplementation	· ANA: Neg<br>· RF: <15<br>· dsDNA: Neg

7.I.1	10	34	NA	F	14, 39		+	+				· Chondromalacia Patellae<br>· Hypermobile knee joints bilaterally<br>· 16-year-old daughter, with GATA2 deficiency and is pre-transplant, has PPP, hypermobile joints, pes planus, chondromalacia patellae, and hypertelorism, but no bifid uvula (7.II.1).	· ANA: 1.4<br>· RF: <10<br>· dsDNA: Neg
8.I.1	21	22	22	F	13, 15	+			+	+	+	· Acute suppurative serositis with necrotizing mesenteric panniculitis<br>· Left leg biopsy showed mixed global panniculitis with eosinophils and neutrophils, without identified bacteria<br>· Pretransplant CT indicated severe cortical bone loss and osteopenia in bilateral distal femurs and proximal tibias<br>· Acute right knee swelling, effusion, complicated by arthrocentesis cell count of 422<br>· Hemophagocytic lymphohistiocytosis<br>· Hypereosinophilia	· ANA: 2.9<br>· RF: <15<br>· CCP: <20<br>· dsDNA: Neg
9.I.1	31	35	36	F	35, 39			+				· Miscarriage	· ANA: Neg<br>· RF: <15<br>· dsDNA: 35
10.I.1	25	51	52	M	08, 39			+				· Mechanical/degenerative vertebral joint disease, complicated by disc herniation<br>· Decreased lumbar flexion, appreciable lumbar vertebral spurs on radiography<br>· Psoriasis treated with topical budesonide	· ANA: 1.1<br>· RF: NA<br>· dsDNA: Neg

11.I.1	<52	52	NA	F	07, 44	+				+	· Two miscarriages (trimester unknown), no children<br>· Arthralgias at shoulders, wrists, neck, back, SI joints, hips, ankles<br>·Chronic back pain associated with morning stiffness, but complicated by disc herniation<br>· Piriformis syndrome	· ANA: Neg<br>· RF: <15<br>· dsDNA: Neg<br>· Lupus anti:coagulant: Positive<br>· PT, PTT wnl<br>· Beta 2 Glycoprotein: Neg<br>· Anticardiolipin antibodies: Neg
12.I.1	40	45	NA	F	37, 38	+		+		+	· Recurrent pericarditis/serositis<br>· Pericardial effusion with lymphocytic and macrophage predominance<br>· Hyperferritinemia (Max 682)<br>· Panniculitis improvement with Anakinra<br>· Arthralgias improved with hydroxychloroquine and Nabumetone	· ANA: 1.6<br>· RF: <15<br>· CCP: <20<br>· dsDNA: Neg<br>· Jo 1: <0.2<br>· RNP: 0.2
13.I.1	11	21	25	F	18, 35	+				+	· Behçet's disease<br>· Treated as teenager with colchicine, etanercept, adalimumab<br>· Maintenance prednisone 2.5mg daily	· ANA: 1.6<br>· RF: <15<br>· dsDNA: Neg
14.I.1	26	25	27	F	41, 51		+		+	+	· Sicca symptoms<br>· Dental clinic Sjogren's evaluation pre-transplant indicated oral and ocular dryness subjective criteria and objective salivary gland involvement via low salivary flow<br>· Salivary gland biopsy Focus score 0, no granulomas, no germinal centers<br>· DEXA scan indicating osteoporosis, lumbar spine	· ANA: 3.5<br>· Anti:PR3: 53.2<br>· Anti:Ro/La: NA<br>· RF: NA<br>· dsDNA: NA<br>· ENA screen:– Neg

15.I.1	32	38	39	F	07,<br>55	+					· Uncharacterized arthralgias bilateral wrists, ankles, feet	· ANA: 0.6 (Neg)<br>· RF: NA<br>· dsDNA: NA
16.I.1	26	22	27	M	14,<br>35	+					· Polyarthralgia with joint swelling bilateral fingers, toes<br>· Controlled by ibuprofen associated with fatigue, malaise<br>· Tiny erosions lateral capitate bone at articulation with trapezoid (R) and mild degenerative first metacarpophalangeal joint (L)	· ANA: Neg<br>· RF: <15<br>· dsDNA: Neg
17.I.1	18	28	28	F	41,<br>51	+	+		+		· Lower back pain, not responsive to NSAID, worse in morning<br>· Headaches<br>· Wrist, elbow, shoulder arthralgias without swelling<br>· DEXA-scan showing osteopenia/osteoporosis	· ANA: Neg<br>· RF: NA<br>· dsDNA: NA
18.I.1	19	21	21	F	07,<br>44					+	· No clear subcutaneous underlying mycobacterial infection	· ANA: 2.1<br>· RF: <15<br>· dsDNA: Neg
19.I.1	14	23	24	F	07,<br>18					+	· Post-transplant biopsy ruled out graft versus host disease, and no clear infectious etiology	· ANA: 1.4<br>· RF: <15<br>· dsDNA: Neg
20.I.1	<4<br>4	44	46	F	40,<br>50	+		+			· Chronic lower back pain, mechanical vs. degenerative<br>· Known mitral valve prolapse on TTE	· ANA: Neg<br>· RF: <15<br>· dsDNA: Neg
21.I.1	31	31	NA	F	13,<br>27	+		+			· Biopsy proven panniculitis with no bacteria identified	· ANA: Neg<br>· RF: <10<br>· dsDNA: Neg

22.I.1	20	21	21	M	15,<br>44			+		· ANA: 0.2<br>· RF: <15<br>· dsDNA: NA
23.I.1	31	32	33	F	27,<br>40		+	+	·Biopsy proven Pyoderma gangrenosum and panniculitis<br>· Treated successfully with colchicine and steroids<br>· DEXA scan indicating osteoporosis/osteopenia and TBS indicating abnormality in trabecular-meshwork microarchitecture<br>· Folliculitis	· ANA: Neg<br>· RF: 18<br>· dsDNA: Neg
24.I.1	35	34	35	F	07,<br>53				· Biopsy proven alopecia areata	· ANA: 1.5<br>· RF: 17<br>· dsDNA: Neg
25.I.1	27	50	51	F	44,<br>50				· Primary biliary cirrhosis	· ANA: 0.4<br>· RF: <15<br>· dsDNA: Neg
26.I.1	<1<br>9	20	20	F	57<br>hom<br>ozyg<br>ous	+			· Bilateral knee synovitis with effusions<br>· Mediastinal lymphadenopathy associated with granulomatous disease concerning for sarcoidosis prior to GATA2 diagnosis<br>· Skin biopsy of LLE indicated medium vessel vasculitis; other skin biopsies indicated non-specific dermal necrosis and mixed inflammatory infiltrate	· ANA: 1<br>· RF: <15<br>· dsDNA: 39

27.I.1	Unclear	Before 46 years old	NA	F	35, 55	+	+	· Panniculitis resolution with prednisone, not with anti-NTM treatment	· ANA: 2.1<br>· RF: <15<br>· dsDNA: NA

Supplemental Table 1b. Summary table of 28 patients with rheumatological manifestations and GATA2 deficiency.

Patient	Additional GATA2 Deficiency Manifestations								
	NTM/PAP/ Recurrent Pneumonias	Deafness/Sensorineural Hearing Loss/Tinnitus	Warts	Disseminated HSV	MDS/AML	Leukopenia/ Thrombocytopenia	Lymphedema/ Emberger Syndrome	Endometriosis/ PCOS/Menorrhagia/Vulvar dysplasia	MonoMAC	Other
1.I.1	+	+			+					
2.I.1	+		+		+		+			· Sarcoidosis vs. PAP<br>· Basal cell carcinoma scalp
3.I.1	+		+	+		+				

4.I.1	+	+	+	+			· Bowen's disease
5.I.1	+				+		· Infectious colitis<br>· CMV-induced HLH
6.I.1	+	+					
6.I.2	+	+	+				
7.I.1						+	· Gastroenteritis

8.I.1								+	
9.I.1				+	+		+		· Pulmonary Granuloma
10.I.1	+		+			+			· Warts treated with imiquimod
11.I.1		+				+	+		· Common variable immunodeficiency<br>· Breast/skin abscesses

12.I.1	+	+	+			+	+		· Granulomatous dermatitis<br>· Necrotizing fasciitis
13.I.1		+		+	+		+		· Asthma<br>· Primary hyperparathyroidism<br>· Pituitary adenoma<br>· Hepatic adenoma
14.I.1			+					+	· Papilledema<br>· Thyroid nodules
15.I.1	+								

16.I.1				+	+			· MRSA infection<br>· Varicella infection
17.I.1			+			+	+	
18.I.1	+	+		+				· Strep pharyngitis
19.I.1	+	+	+		+	+		
20.I.1	+							
21.I.1	+		+		+			
22.I.1			+		+			· Diffuse lymphadenopathy

23.I.1	+	+	+			+		
24.I.1	+					+	+	·Cryptococcal meningitis<br>· Aspergillus infection
25.I.1	+				+			· Disseminated M. kansasii infections<br>· Bilateral iliopsoas abscesses
26.I.1	+			+				
27.I.1	+	+					+	· Disseminated M. kansasii infection

Supplemental Table 2. Literature review results.

Number of patients with GATA2 deficiency and rheumatological manifestations described and summary of manifestations described.

Study	Number of Patients with GATA2 deficiency and Rheumatological Manifestations	Manifestations Described									
		Arthralgias/Arthritis	PPP/Hyperextensibility	Psoriasis	Osteopenia/Early-Onset Osteoporosis	Panniculitis	Livido Reticularis	Erythema Nodosum	Pyoderma Gangrenosum	Autoimmune/Inflammatory Manifestations	Other/Descriptions
Moraes-Fontes, M. F., et al. (2019)	1			+							· Asthma and hypothyroidisms as a child
Polat, A., et al. (2018)	1					+		+			· Sclerodermiform lesions on the legs, panniculitis on the upper limbs and upper limb, biopsy proven erythema nodosum with negative mycobacterium cultures.
Vinh, D. C., et al. (2010)	10					+		+		+	· In 6 patients, multiple inflammatory nodules demonstrating panniculitis or granulomatous inflammation without microorganisms were observed<br>· One patient, severe postpartum rash diagnosed w/histology as discoid lupus, CVID diagnosis, erythema nodosum.<br>· One patient, lupus-like pattern<br>· One patient, primary biliary cholangitis-like pattern<br>· One patient, multiple sclerosis-like syndrome
Damian, L., et al. (2018)	1					+					
Rae, W., et al. (2017)	1									+	· Autoimmune hemolytic anemia, immune thrombocytopenic purpura, pulmonary fibrosis treated with rituximab, sirolimus.

Webb, G., et al. (2016)	1				+	· Autoimmune hepatitis
Johnson, J. A., et al. (2015)	1					· Suppurative lymphadenitis, acute suppurative serositis identified by skin biopsy, and vasculitic-appearing ulcerations on knees, hands, and shoulders.
Dickinson, R. E., et al. (2014)	8	+	+		+	· Autoimmune related findings described as arthritis, panniculitis, or autoimmune cytopenia
Camargo, J. F., et al. (2013)	1			+		· Biopsy proven erythema nodosum, with negative bacterial, fungal, and AFB cultures
Ishida, H., et al. (2012)	1		+			· Recurrent panniculitis
Bigley, V., et al. (2011)	4	+	+	+		· Autoimmune related findings described as erythema nodosum/panniculitis or arthritis

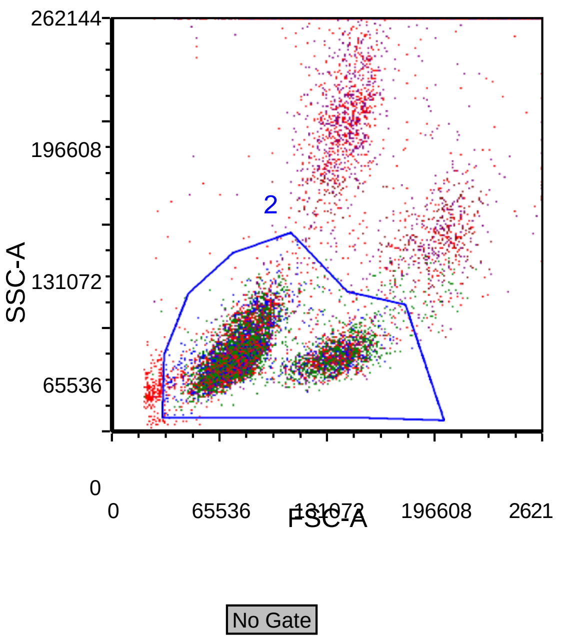

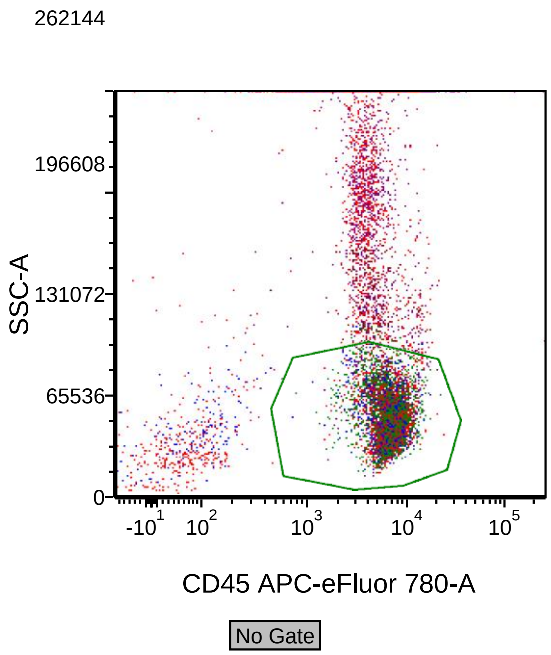

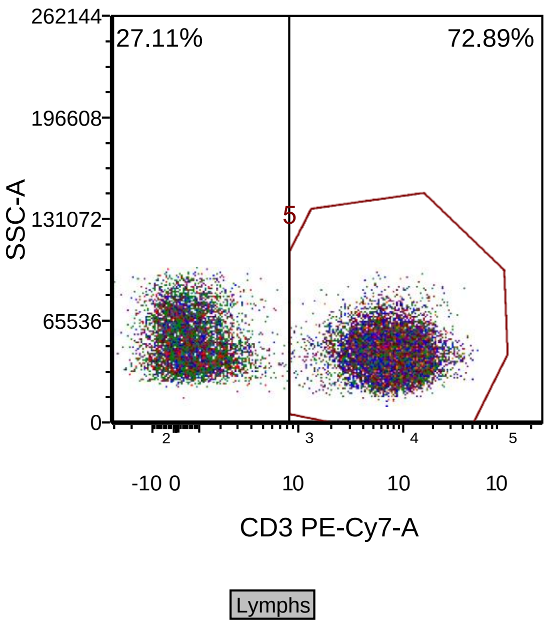

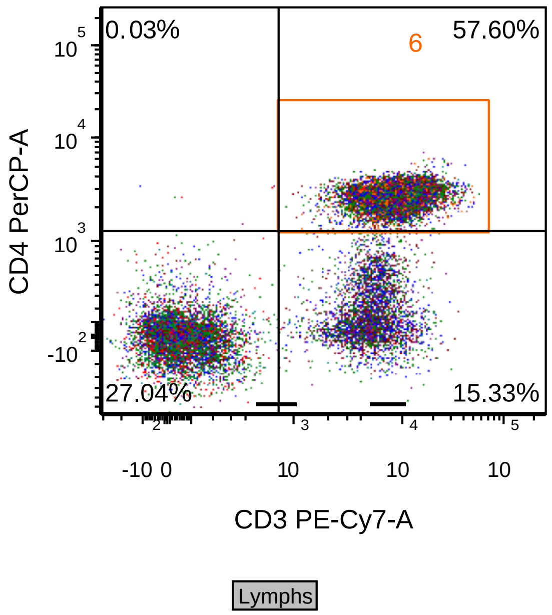

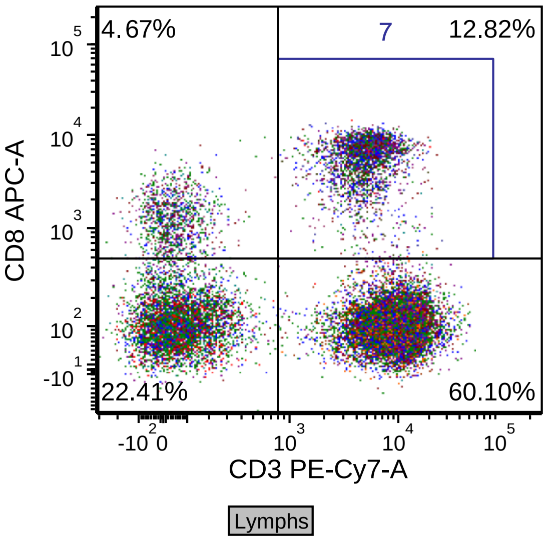

Supplemental Figure 1. Lymphocyte phenotype gating strategy.
Lymphocytes were defined based on scatter and CD45, plus CD3, CD4, CD8 expression.

Supplemental Methods: Search strategies for literature review

Database: PubMed/MEDLINE
Vendor: NLM
Date: October 15, 2019
Limits: English language only English[lang]; MEDLINE only medline [sb]

Total = 52
Total & English language = 51
Total & English language & MEDLINE = 48

("GATA2 Deficiency"[Mesh] OR "GATA2 Transcription Factor"[Mesh] OR GATA2[tiab] OR "GATA 2"[tiab] OR "GATA2 Deficiencies"[tw] OR "GATA2 deficiency"[tw] OR "GATA 2 deficiency"[tw] OR "MonoMac Syndrome"[tw] OR "GATA2 Haploinsufficiency"[tw] OR "Natural Killer Cell Deficiency"[tw] OR "Natural Killer Cell Deficiencies"[tw] OR "Emberger Syndrome"[tw] OR "Primary Lymphedema with Myelodysplasia"[tw] OR "Myelodysplastic Syndrome Acute Myeloid Leukemia"[tw] OR "GATA Binding Protein 2"[tw] OR "transcription factor GATA2"[tw] OR "transcription factor GATA 2"[tw])

AND

("Osteoarthritis"[Mesh] OR "Spondylarthropathies"[Mesh] OR "Sacroiliitis"[Mesh] OR "Enthesopathy"[Mesh] OR "Fractures, Bone"[Mesh] OR "Psoriasis"[Mesh] OR "Inflammatory Bowel Diseases"[Mesh] OR "Crohn Disease"[Mesh] OR "Colitis, Ulcerative"[Mesh] OR "Inflammation"[Mesh] OR "Spondylitis, Ankylosing"[Mesh] OR "Low Back Pain"[Mesh] OR osteoarthritis[tiab] OR osteoarthritic[tiab] OR "degenerative arthritis"[tiab] OR spondylarthropathies[tiab] OR spondylarthropathy[tiab] OR sacroiliitis[tiab] OR enthesopathy[tiab] OR enthesopathies[tiab] OR fracture[tiab] OR fractures[tiab] OR "broken bone"[tiab] OR "broken bones"[tiab] OR musculoskeletal[tiab] OR psoriasis[tiab] OR inflammation[tiab] OR "inflammatory bowel disease"[tiab] OR "crohn disease"[tiab] OR "crohn's disease"[tiab] OR "ulcerative colitis"[tiab] OR skeletal[tiab] OR "Ankylosing spondylitis"[tiab] OR "Ankylosing Spondylarthritis"[tiab] OR "Bechterew Disease"[tiab] OR "Marie Struempell Disease"[tiab] OR "Marie Strumpell Spondylitis"[tiab] OR "low back pain"[tiab] OR "low back pains"[tiab] OR lumbago[tiab])

[tw] = text word field
[tiab] = title and abstract fields
[mesh] = Medical Subject Heading field

Database: Embase.com
Vendor: Elsevier
Date: October 15, 2019
Limits: English language [english]/lim; Publication Type: NOT ([conference abstract]/lim OR [conference paper]/lim OR [conference review]/lim)

Total = 414
Total & English language = 410
Total & English language & NOT conference proceedings = 261

('GATA2 deficiency'/exp OR 'transcription factor GATA 2'/exp OR GATA2:ti,ab OR "GATA 2":ti,ab OR "GATA2 Deficiencies":ti,ab OR "GATA2 deficiency":ti,ab OR "GATA 2 deficiency":ti,ab OR "MonoMac Syndrome":ti,ab OR "GATA2 Haploinsufficiency":ti,ab OR "Natural Killer Cell Deficiency":ti,ab OR "Natural Killer Cell Deficiencies":ti,ab OR "Emberger Syndrome":ti,ab OR "Primary Lymphedema with Myelodysplasia":ti,ab OR "Myelodysplastic Syndrome Acute Myeloid Leukemia":ti,ab OR "GATA Binding Protein 2":ti,ab OR "transcription factor GATA2":ti,ab OR "transcription factor GATA 2":ti,ab) AND ('osteoarthritis'/exp OR 'ankylosing spondylitis'/exp OR 'spondyloarthropathy'/exp OR 'fracture'/exp OR 'bone injury'/exp OR 'low back pain'/exp OR 'sacroiliitis'/exp OR 'enthesopathy'/exp OR 'psoriasis'/exp OR 'inflammatory bowel disease'/exp OR 'inflammation'/exp OR 'Crohn disease'/exp OR 'ulcerative colitis'/exp OR osteoarthritis:ti,ab OR osteoarthritic:ti,ab OR "degenerative arthritis":ti,ab OR spondylarthropathies:ti,ab OR spondylarthropathy:ti,ab OR sacroiliitis:ti,ab OR enthesopathy:ti,ab OR enthesopathies:ti,ab OR fracture:ti,ab OR fractures:ti,ab OR "broken bone":ti,ab OR "broken bones":ti,ab OR musculoskeletal:ti,ab OR psoriasis:ti,ab OR inflammation:ti,ab OR "inflammatory bowel disease":ti,ab OR "crohn disease":ti,ab OR "crohns disease":ti,ab OR "ulcerative colitis":ti,ab OR skeletal:ti,ab OR "Ankylosing spondylitis":ti,ab OR "Ankylosing Spondylarthritis":ti,ab OR "Bechterew Disease":ti,ab OR "Marie Struempell Disease":ti,ab OR "Marie Strumpell Spondylitis":ti,ab OR "low back pain":ti,ab OR "low back pains":ti,ab OR lumbago:ti,ab)

/exp = explode the EMTREE term
:ti,ab = title and abstract fields

Database: Cochrane Library: CENTRAL & Database of Systematic Reviews Vendor: Wiley & Sons
Date: October 15, 2019
Limits: None

Total = 5 Trials

#1 ([mh "GATA2 Deficiency"] OR [mh "GATA2 Transcription Factor"])

#2 (GATA2 OR "GATA 2" OR "GATA2 Deficiencies" OR "GATA2 deficiency" OR "GATA 2 deficiency" OR "MonoMac Syndrome" OR "GATA2 Haploinsufficiency" OR "Natural Killer Cell Deficiency" OR "Natural Killer Cell Deficiencies" OR "Emberger Syndrome" OR "Primary Lymphedema with Myelodysplasia" OR "Myelodysplastic Syndrome Acute Myeloid Leukemia" OR "GATA Binding Protein 2" OR "transcription factor GATA2" OR "transcription factor GATA2"):ti,ab,kw

#3 ([mh "Osteoarthritis"] OR [mh "Spondylarthropathies"] OR [mh "Sacroiliitis"] OR [mh "Enthesopathy"] OR [mh "Fractures, Bone"] OR [mh "Psoriasis"] OR [mh "Inflammatory Bowel Diseases"] OR [mh "Crohn Disease"] OR [mh "Colitis, Ulcerative"] OR [mh "Inflammation"] OR [mh "Spondylitis, Ankylosing"] OR [mh "Low Back Pain"])

#4 (osteoarthritis OR osteoarthritic OR "degenerative arthritis" OR spondylarthropathies OR spondylarthropathy ORsacroiliitis OR enthesopathy OR enthesopathies OR fracture OR fractures OR "broken bone" OR "broken bones" OR musculoskeletal OR psoriasis OR inflammation OR "inflammatory bowel disease" OR "crohn disease" OR "crohn's disease" OR "ulcerative colitis" OR skeletal OR "Ankylosing spondylitis" OR "Ankylosing Spondylarthritis" OR "Bechterew Disease" OR "Marie Struempell Disease" OR "Marie Strumpell Spondylitis" OR "low back pain" OR "low back pains" ORlumbago):ti,ab,kw

#5 #1 OR #2

#6 #3 OR #4

#7 #5 AND #6

mh = Medical Subject Heading field
:ti,ab,kw = title, abstract and keyword fields

Database: Scopus
Vendor: Elsevier
Date: October 15, 2019
Limits: English language only AND (LIMIT-TO (LANGUAGE , "English"); Title-Abstract-Keyword fields TITLE-ABS-KEY; Document Types (excluding Conference Paper & Book Chapter (EXCLUDE (DOCTYPE , "cp") OR EXCLUDE (DOCTYPE , "ch"))

Total = 130
Total & English language = 128
Total & English language & Document Types: Article, Review, Short Survey, Letter, Note = 124

TITLE-ABS-KEY((GATA2 OR {GATA 2} OR {MonoMac Syndrome} OR {Natural Killer Cell Deficiency} OR {Natural Killer Cell Deficiencies} OR {Emberger Syndrome} OR {Primary Lymphedema with Myelodysplasia} OR {Myelodysplastic Syndrome Acute Myeloid Leukemia})

AND

(Osteoarthritis OR Spondylarthropathies OR Sacroiliitis OR Enthesopathy OR Psoriasis OR {Inflammatory Bowel Diseases} OR {Crohn Disease} OR {ulcerative Colitis} OR Inflammation OR {Ankylosing Spondylitis} OR {Low Back Pain} OR osteoarthritic OR {degenerative arthritis} OR spondylarthropathy OR enthesopathies OR fracture OR fractures OR {broken bone} OR {broken bones} OR musculoskeletal OR {crohn's disease} OR skeletal OR {Ankylosing Spondylarthritis} OR {Bechterew Disease} OR {Marie Struempell Disease} OR {Marie Strumpell Spondylitis} OR {low back pains} OR lumbago))

TITLE-ABS-KEY ((gata2 OR {GATA 2} OR {MonoMac Syndrome} OR {Natural Killer Cell Deficiency} OR {Natural Killer Cell Deficiencies} OR {Emberger Syndrome} OR {Primary Lymphedema with Myelodysplasia} OR {Myelodysplastic Syndrome Acute Myeloid Leukemia}) AND (osteoarthritis OR spondylarthropathies OR sacroiliitis OR enthesopathy OR psoriasis OR {Inflammatory Bowel Diseases} OR {Crohn Disease} OR {ulcerative Colitis} OR inflammation OR {Ankylosing Spondylitis} OR {Low Back Pain} OR osteoarthritic OR {degenerative arthritis} OR spondylarthropathy OR enthesopathies OR fracture OR fractures OR {broken bone} OR {broken bones} OR musculoskeletal OR {crohn's disease} OR skeletal OR {Ankylosing Spondylarthritis} OR {Bechterew Disease} OR {Marie Struempell Disease} OR {Marie Strumpell Spondylitis} OR {low back pains} OR lumbago)) AND (LIMIT-TO (LANGUAGE , "English")) AND (EXCLUDE (DOCTYPE , "cp") OR EXCLUDE (DOCTYPE , "ch"))
